# To Evaluate Cytotoxicity of Resin-based Restorative Materials on Human Lymphocytes by Trypan Blue Exclusion Test: An *in vitro* Study

**DOI:** 10.5005/jp-journals-10005-1070

**Published:** 2010-09-15

**Authors:** Vinay Kumar Srivastava, Rajat Kumar Singh, SN Malhotra, Aditi Singh

**Affiliations:** 1 Professor and Head, Department of Pedodontics and Preventive Dentistry, Saraswati Dental College, Lucknow, Uttar Pradesh India; 2 MDS (PG-III), Department of Pedodontics and Preventive Dentistry, Saraswati Dental College, Lucknow, Uttar Pradesh, India; 3 Professor and Head, Department of General Pathology, Saraswati Dental College, Lucknow, Uttar Pradesh, India; 4 Reader, Department of Microbiology, Saraswati Dental College, Lucknow, Uttar Pradesh, India

**Keywords:** Nanocomposite, Cytotoxicity, HISEP LSM, Trypan blue.

## Abstract

The cytotoxicity of a resin-based material can be evaluated on isolated human lymphocyte. Since resin-based dental materials have been used with increasing frequency in anterior and posterior teeth restorations, the uncured resin monomers are leached out from the restorations and diffuses into the dentine and ultimately hampers the odontoblastic layers of pulp as well as gingiva. It is also reaches into the saliva and circulatory blood. The study evaluates and compares the relative cytotoxicity of resin-based dental materials at different time interval, i.e. 24, 48, and 72 hours on human lymphocyte by Trypan blue exclusion method. All resins were found to be cytotoxic to human lymphocyte. Resin samples cytotoxicity was the highest in first 24 hours followed by 48 and 72 hours.

## INTRODUCTION

Resins composites have been used as posterior restorative material with increasing frequency because of demand for both esthetic restorations and worries about adverse effects of mercury from amalgam (Sweeney et al 2002).^1^ Chronic pulpitis were reported from the use of dental composites in cavities that have not been properly protected. Thus, investigators such as Stanley et al (1978)^2^ and Suarez et al (1978)^3^ have recommended that composites be classified as toxic. Baume and Fiore-Donna (1968)^4^ stated that this pulp reaction to composite was mild, if there was 1 mm or more of remaining dentine, however decayed teeth displayed more severe reactions. Chemicals and bacteria have been proposed as explanations for pulpal irritation from dental composites. Fujisawa et al (1999)^3^ proposed that chemical irritation resulted from the hemolytic potency of BIS-GMA and other acrylates and vinyl monomers caused by the highly hydrophobic nature of the composites.

Hanks et al (2000) proposed that the chemical irritation of composites was the result of the effect of extractants from the composites. However, Stanley et al in 1979^2^ showed that placing ingredients on dentine showed no significant response. The cytotoxicity of dental composites has been studied using different methods.^5^ The toxicity of dental material can be evaluated by *in vitro* test and through clinical studies in humans. Unfortunately, little is known about the toxicity of resin-based tooth colored restorative materials at different time intervals keeping the light source constant.

In the study, three different polymerized and unpoly-merized resins-based tooth colored materials were evaluated for possible cytotoxicity at three different time intervals and results were compared with each other. When evaluating the cytotoxicity effect, cell viability was evaluated using Trypan blue exclusion test.

## MATERIALS AND METHODS

### Materials

Hisep- LSM (1.0770 gm/ml), trypan blue (0.4 % in 0.81% sodium chloride and 0.06% potassium phosphate), normal physiological saline, compound microscope (Olympus BH Tokyo, Japan), electronic cell counter (Cell tech 30), esthete X flow flowable composite (Dentsply Co.), Synergy D_6 _nanocomposite (Coltene Whaledent), Dyract compomer (Dentsply), QHL 750 Halogen light cure unit, polyethylene Molds with diameter 2 × 2 mm, sterile test tubes, test tube stand, disposable syringes (5 ml and 2 ml), EDTA, glass slides, cover slips and aliquots.

### Methods

Isolation of Lymphocytes

**Fig. 1:**
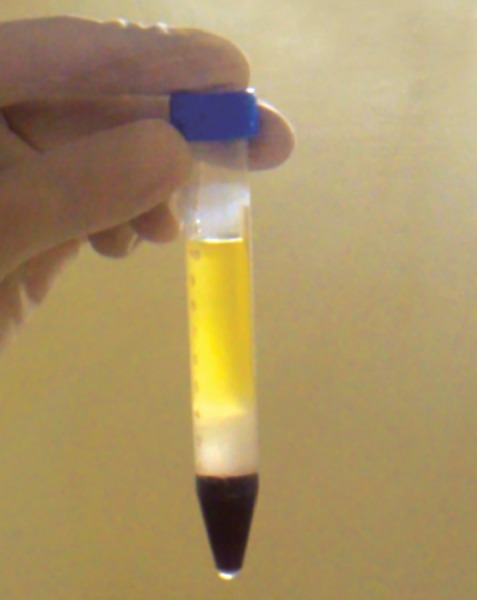
Lymphocyte layer at the interphase of plasma and HISEP-LSM

*Procedure:* 2.5 ml of HISEP-LSM (Iso-osmotic, low viscosity medium containing polysucrose and sodium diatrizoate adjusted to density of 1.0770 gm/ml) was transferred aseptically to a 15 ml autoclaved centrifugal tube. About 4 ml EDTA treated human blood was collected aseptically from the donor and diluted 1:1 with 4 ml of physiological saline. 7.5 ml of diluted blood was transferred along the walls of the centrifugal tube containing 2.5 ml HISEP-LSM gently. The tube containing HISEP-LSM and diluted blood was centrifuged at 600 rpm at room temperature for 40 minutes. Centrifugation has sedimented erythrocytes and polynuclear leukocytes, mononuclear lymphocytes have formed an interphase above HISEP-LSM (Fig. 1).

**Fig. 2:**
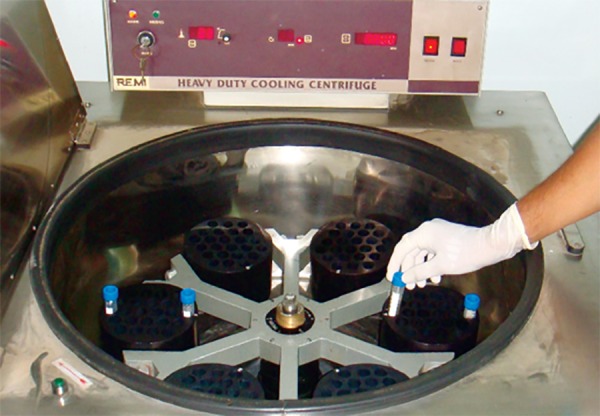
Centrifugation of overlaid blood at 600 rpm

**Fig. 3:**
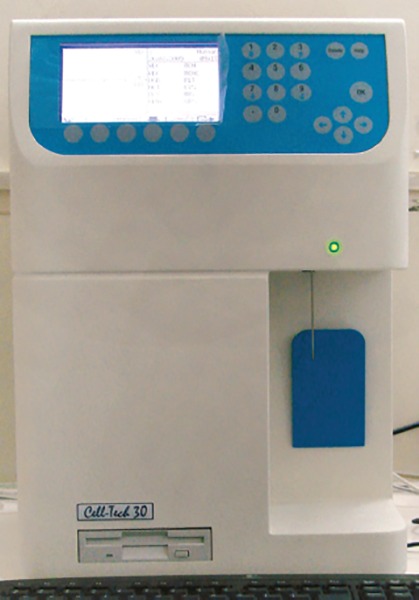
Electronic cell counter

Most of the plasma and platelet containing supernatant above the interphase band was aspirated (granulocytes and erythrocytes will be in the red pellet) then carefully aspirate the lymphocyte layer above with half of the HISEP-LSM layer below it and transfer it to a clean centrifuge tube. Add an equal amount of physiological saline to the lymphocyte layer in the centrifugal tube and mix by gentle aspiration. Centrifuge for 10 minutes at room temperature at a speed of 600 rpm (Fig. 2). This washing with physiological saline removes HISEP-LSM and reduces the number of platelets. The cell pellet after discarding the supernatant was gently resuspended in 2 mg of RPMI medium. The cell counting was done using electronic cell counter (Fig. 3) and viability checked by supravital staining with 0.1% Trypan blue which was kept as a negative control.

### Preparation of Composite

**Fig. 4:**
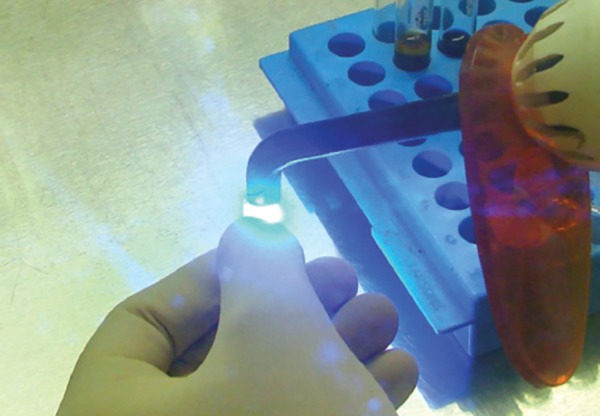
Resin material curing with hallogen light at the distance of 1 mm from the mold

A polyethylene mold with diameter 2 × 2 mm was taken to standardize the amount of composite used in the experiment. One set of the unpolymerized composites was filled in the polyethylene mold and mechanically pressed under mylar strip to remove the excess material. These were directly introduced into the lymphocyte cultures as control group. The other set of composite samples was mechanically pressed within the polyethylene mold under Mylar strip. After load removing, the top surfaces of the specimens were light cured at a distance of 1 mm from the curing tip to the specimens (Fig. 4).

**Fig. 5:**
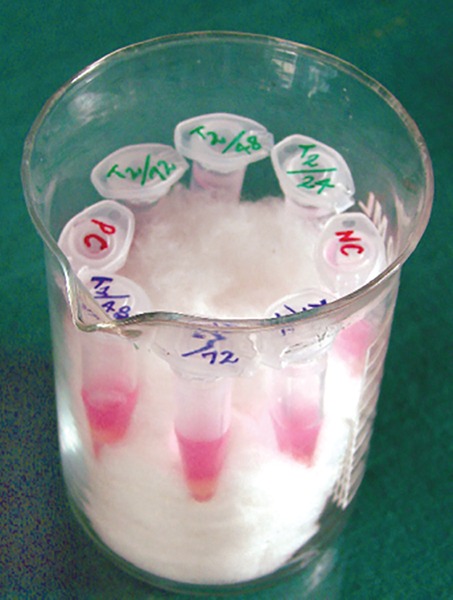
Specimen kept in Aliquots (aseptic sealed small tube) in incubator containing 5% CO_2_

**Fig. 6:**
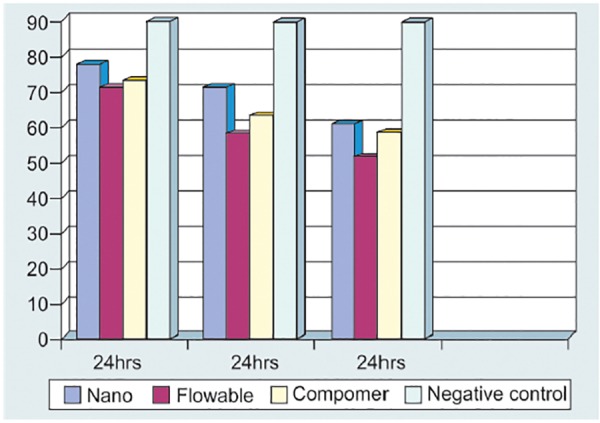
Bar diagram depicted a comparative mean percentage viability of human lymphocyte at different incubation times, i.e. 24, 48, and 72 hours with resin materials

**Fig. 7:**
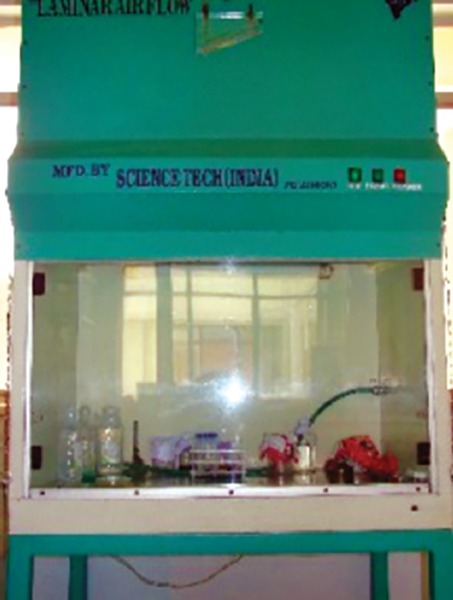
Procedures have been done in Laminar airflow chamber to avoid any contamination

In the study, conventional quartz-tungsten-halogen (QTH) lamps were used, which emit light within the wavelength range of 400 to 500 nanometers. The intensity of the light polymerization unit was regularly monitored after every third curing to ensure quality composite restorations. After each three specimen curing, the light intensity was monitored by a radiometer. Thereafter, the polymerized composites were separated from Mylar strips, mold in aseptical condition and finally introduced into the lymphocyte cultures. These cultures were kept in three sets of Aliquots* (Fig. 5) for 24, 48 and 72 hours at 37°C in a 5% CO_2_ atmosphere to maintain sterile condition (Fig. 6). This procedure was repeated with all three resin-based tooth colored materials. Equal number of lymphocytes was incubated alongside without any composite as a control group. Procedures were done in laminar airflow chamber to maintain aseptic condition (Fig. 7).

### Cytotoxicity Testing

**Fig. 8:**
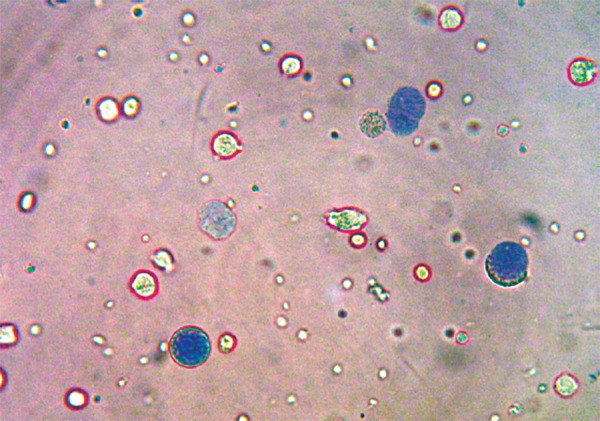
Human lymphocytes cell viability counts is inversibly proportional to the cytotoxicity of composite materials. Unstained cells with Trypan blue dye are counted as viable and stained was counted as non-viable

Assessing cell membrane integrity is one of the most common ways to measure cell viability and cytotoxic effects. Compounds that have cytotoxic effects often compromise cell membrane integrity. Vital dye, such as Trypan blue is normally excluded from the inside of healthy cells, however if the cell membrane integrity has been compromised, dye freely crosses the membrane and stains intracellular components (Fig. 8).

### Procedure

**Fig. 9:**
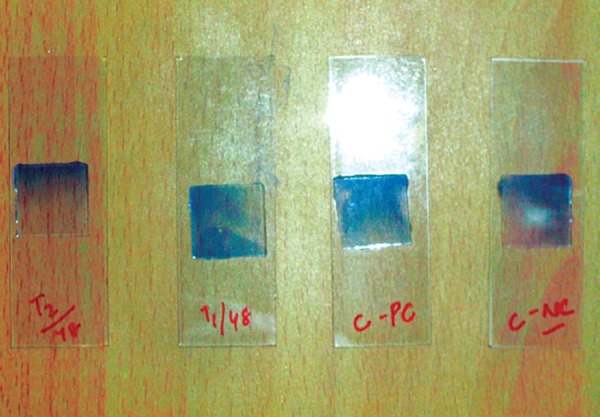
20 μl of suspension was mixed with 20 μl of 0.4% Trypan blue and observed under CH-20i light microscope

The set of the culture containing composite was removed after 24, 48, and 72 hours and centrifuged at 600 rpm for 10 minutes and 4.5 ml of supernatant was removed. The pellet was gently resuspended and 20 μl of suspension was mixed with 20 μl of 0.4% Trypan blue to check the cell viability. A drop of dye and cell suspension was taken on a clean glass slide, covered by a cover slip (Fig. 9) and analyzed under CH-20i light microscope (Olympus, Tokyo, Japan) using 40X magnification. For each lymphocyte culture (for each sample of composite cured using a specific curing program), 500 lymphocytes were analyzed counting the unstained viable cells. Similar four more slides were prepared as above for each group to minimize the manual error. The control groups were also treated after each incubation period in the same way as mentioned above.

## RESULTS

### Effect of Different Materials (Fig. 10 and Table 1)

Among different materials (under different variable cure conditions and treatment times), the mean percentage viability of material Nano was found to be maximum with a mean value of 69.07 ± 11.85% (range 43-85%) while that of flowable was found to be minimum, i.e. 59.24 ± 13.60% (range 29-80%). The mean percentage viability of negative control (without any material) was found to be 89.39 ± 2.17% (range 87-95%).

On comparing the data statistically, it was found that mean percentage viability of Nano was significantly higher as compared to flowable (p < 0.001). Although the mean percentage viability of Nano was higher as compared to Compomer, yet there was no statistically significant difference between the two (p = 0.076). On comparing the percentage viability of flowable with Compomer, the mean percentage viability of Compomer was found to be higher as compared to flowable yet no statistically significant difference between the two could be seen (p = 0.095). On comparing the different study groups with negative control group, the mean percentage viability of all three groups was found to be significantly lower as compared to negative control group (p < 0.001).

**Fig. 10:**
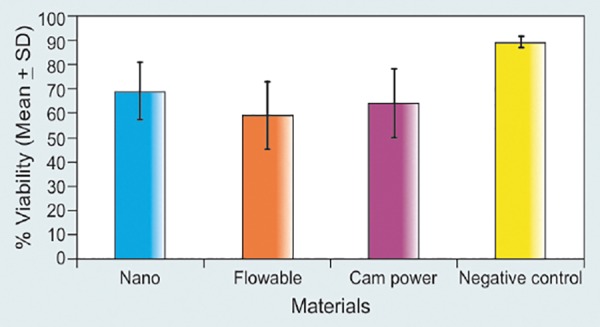
The above bar diagram showed relative mean percentage viability of lymphocyte with different resin-based restorative materials

### Effect of Time

A statistically significant fall in percentage viability was seen with increase in incubation time (p < 0.001). The percentage viability was minimum in first 24 hours for all three resin-based tooth colored restorative materials followed by 48 and 72 hours respectively.

**Table 1:** **Table 1:** Showed percentage viability of lymphocyte with different resin materials under different incubation times by Trypan blue exclusion test

*Material*		*Trypan blue*				*Statistical significance*			
		Mean		SD		t		^p^	
24 hours									
• Nano		77.00		6.83		0.245		0.808	
• Flowable composite		70.47		9.00		-2.113		0.044	
• Compomer		72.33		9.98		0.252		0.803	
After 48 hours									
• Nano		70.20		11.22		0.802		0.429	
• Flowable composite		56.80		11.49		-1.612		0.118	
• Compomer		62.53		13.45		1.315		0.199	
After 72 hours									
• Nano		60.00		10.56		1.422		0.166	
• Flowable composite		50.47		12.03		-1.695		0.101	
• Compomer		57.60		14.75		1.245		0.224	
• Negative control		89.39		2.17		2.186		0.036	

## DISCUSSION

Human lymphocytes were used for cytotoxicity testing in the study because they are sensitive cells that can be isolated as pure population from blood and cultured in normal culture medium. The culture used in the study was RPMI medium. RPMI is a basal medium consisting of vitamins, amino acids, salts, glucose, glutathione and a pH indicator. Composite material, however has been shown to elicit a chronic inflammatory response *in vivo* (Nasjleti et al, 1983)^6^ to be cytotoxic in cell culture (Hensten-Pettersen and Helgeland, 1977, 1981; Mjor, 1977; Wennberg and Hensten-Pettersen, 1981; Kasten et al, 1982), to be potentially allergenic (Nathanson and Lockart, 1979; Kallus et al, 1983; School, 1991), and to inhibit RNA synthesis (Caughman et al, 1990).

Composite restorative materials are a mixture of polymerized resin components reinforced by inorganic fillers (Peutzfeldt, 1997; Rueggeberg, 2002).^7^ Several studies have showed that monomers and other components were released from these materials into the oral environment even after polymerization. Among some 30 chemicals, the monomer 2-hydroxyethyl methacrylate (HEMA) and the co-monomer triethylene glycol dimethacrylate (TEGDMA) were detected (Santerre et al, 2001; Michelsen et al, 2003).^8^ Both HEMA and TEGDMA may diffuse through dentin in sufficient concentrations to cause cellular damage (Bouillaguet et al, 1996; Hume and Gerzina, 1996).^8^ It has been estimated that the concentrations of HEMA and TEGDMA available from, for instance, dentinal adhesives would be in the millimolar range after diffusion through the dentin layer. HEMA leaching from dentin adhesives might reach concentrations as high as 1.5-8 mmol/L in the pulp. Likewise, the TEGDMA concentrations reaching the pulp after diffusion across dentin in deep cavities could be in the range of 4 mmol/L (Bouillaguet et al, 1996; Noda et al, 2002).^7^

The materials used in the study were Esthete X flow (flowable composite), synergy D_6_ (nanocomposite), and a Dyract (compomer) in which flowable composite showed the maximum cytotoxicity, which was probably due to more resin content and less of filler load. Compomer and nanocomposite were less toxic to human lymphocytes compared to flowable. As per the study, flowable composite should be used where there is sufficient amount of dentine thickness left. It should be avoided in fractured restorations which are near to the pulp as it is left with most unreacted monomers that can damage the pulp. Compomers can be the material of choice in deciduous teeth as it showed less cytotoxic and having advantage of fluoride release but, as a base it should never be used in deep cavities as this material is also cytotoxic to some extent. Nanocomposite according to the present data proved to be the safest amongst the entire three tooth colored restorative materials but this material also releases some uncured base monomers after final set.

Taken together cytotoxicity tests done in this study proved that cytotoxic substances leach from resin-based tooth colored restorative materials, particularly when unset or short after polymerization. Comparing studies between resin-based composites using equivalent test systems revealed a similar cytotoxicity pattern for all the materials. The general risk for presently available resin- based composites and adhesives are such that their withdrawal from the market is generally not justified. Therefore, now it is necessary to investigate possible damage of adequate target cells in patient and operator in consecutive studies.

## CONCLUSION

The results of the study revealed that:

1. A flowable composite is most cytotoxic followed by compomer and nanocomposite.
2. Materials were most cytotoxic in initial 24 hours which continued but lowered further in 48 and 72 hours respectively.
